# AtANN1 and AtANN2 are involved in phototropism of etiolated hypocotyls of *Arabidopsis* by regulating auxin distribution

**DOI:** 10.1093/aobpla/plab075

**Published:** 2021-12-17

**Authors:** Xiaoxu Wang, Lijuan Han, Hongmin Yin, Zhenping Zhao, Huishu Cao, Zhonglin Shang, Erfang Kang

**Affiliations:** 1 Key Laboratory of Molecular and Cellular Biology of the Ministry of Education, College of Life Sciences, Hebei Normal University, Shijiazhuang 050024, China; 2 Department of Agricultural and Animal Engineering, Cangzhou Vocation College of Technology, Cangzhou 061001, China

**Keywords:** *Arabidopsis thaliana*, AtANN1, AtANN2, auxin, phototropism

## Abstract

Phototropism is an essential response in some plant organs and features several signalling molecules involved in either photo-sensing or post-sensing responses. Annexins are involved in regulating plant growth and its responses to various stimuli. Here, we provide novel data showing that two members of the Annexin family in *Arabidopsis thaliana*, AtANN1 and AtANN2, may be involved in the phototropism of etiolated hypocotyls. In wild type, unilateral blue light (BL) induced a strong phototropic response, while red light (RL) only induced a weak response. The responses of single- or double-null mutants of the two annexins, including *atann1*, *atann2* and *atann1/atann2*, were significantly weaker than those observed in wild type, indicating the involvement of AtANN1 and AtANN2 in BL-induced phototropism. Unilateral BL induced asymmetric distribution of DR5-GFP and PIN3-GFP fluorescence in hypocotyls; notably, fluorescent intensity on the shaded side was markedly stronger than that on the illuminated side. In etiolated *atann1*, *atann2* or *atann1*/*atann2* hypocotyls, unilateral BL-induced asymmetric distributions of DR5-GFP and PIN3-GFP were weakened or impaired. Herein, we suggest that during hypocotyls phototropic response, AtANN1 and AtANN2 may be involved in BL-stimulated signalling by regulating PIN3-charged auxin transport.

## Teaser

Annexins are calcium- and cell membrane-binding proteins. Here, *Arabidopsis* seeds of null mutants of two annexins, AtANN1 and AtANN2, and their wild type (Col-0) were germinated in darkness, then the etiolated seedlings were illuminated by unilateral blue light (BL). In Col-0 seedlings, unilateral BL induced phototropic bending growth and asymmetric distribution of auxin or PIN3 (an auxin transporter) in hypocotyl cells. These responses were much weaker or even disappeared in mutant seedlings, suggesting that the two annexins may play essential role in unilateral BL-induced auxin transport and bending growth.

## Introduction

Responding to unevenly existing physical and/or chemical stimuli (e.g. light, gravity, moisture, chemicals and water), growth rate and direction of some plant organs may be altered. The stimuli-induced positive or negative tropic bending of plant organs, including phototropism, gravitropism, hydrotropism and obstacle avoidance, play essential roles in plants’ adaption to changing environment (Han *et al.* 2021).

Unidirectional light illumination usually results in positive tropism in stems and negative tropism in roots. Phototropism is accomplished by a series of signalling events in plant cells, which initiated by stimulation of light with certain wavelengths (Fankhauser and Christie 2015; Harmer and Brooks 2018). Blue light (BL) and red light (RL) have been shown to induce phototropism, in which BL is regarded as the most important trigger of phototropism (Hohm *et al.* 2013; Sullivan *et al.* 2016; Zhang *et al.* 2017). Blue light receptor, especially phototropin, plays essential roles in light perception and subsequent asymmetric cell elongation. In *Arabidopsis thaliana*, BL stimulates auto-phosphorylation of PHOT1. Then PHOT1 phosphorylates several downstream signalling components as a light-activated protein kinase, including ATP-binding cassette B19 (ABCB19) and phytochrome kinase substrate 4 (PKS4). The phosphorylation leads deactivation of these proteins. Activated PHOT1 also leads the de-phosphorylation of non-phototropic hypocotyl 3 (NPH3), then NPH3 dissociates from membrane to form cytosolic aggregates (Ding *et al.* 2011; Zhang *et al.* 2017; Sullivan *et al.* 2019). The interaction between NPH3 and Cullin 3-RBX-E3 (CLR3) complex leads ubiquitination and internalization of PHOT1 and several signalling components, some of which are essential for auxin polar transport (Roberts *et al.* 2011). When BL intensity increases, another BL receptor (PHOT2) was found to mediate the relocation or stabilization of NPH3 in plasma membrane (PM) (Zhao *et al.* 2018). Following these early signalling events, which have different intensity in illuminated- and shaded-side cells, asymmetric auxin distribution which results from auxin synthesis and lateral transport leads bending of plant organs (de Wit *et al.* 2016; Christie *et al.* 2018; Harmer and Brooks 2018; Han *et al.* 2021).

Several auxin transporters have been shown to participate BL-stimulated auxin polar transport. For instance, the PIN-FORMED (PIN) proteins and ABCB19 charge auxin efflux and are involved in auxin circulation and redistribution (Goyal *et al.* 2013; Liscum *et al.* 2014; Fankhauser and Christie 2015; Han *et al.* 2021). Under unilateral BL illumination, PHOT1-induced deactivation of ABCB19 was stronger in the illuminated-side cells than in the shaded-side cells; more auxin will be transported along the shaded-side cells from tip to basal part of young seedlings (Christie *et al.* 2011). Auxin transporters’ circulation and relocation lead asymmetric auxin transport as well. In dark-grown *Arabidopsis* seedlings, PIN3 located evenly in different parts of hypocotyls. Uneven or unilateral illumination induced a polar localization of PIN3 (PIN3 located mainly in endodermal cells at the shaded side), and a sustained auxin gradient across the hypocotyl (Ding *et al.* 2011). Intracellular vesicle trafficking may be involved in auxin transporter circulation, since brefeldin A (BFA) strongly inhibited the trafficking of PINs (Titapiwatanakun and Murphy 2009; Nishimura *et al.* 2012).

As Ca^2+^- and phospholipid-binding proteins, which located in PM or inner membranes, as well as cytoplasm, annexins play multiple roles in plant growth, development and stress responses. Annexins are involved in vegetative growth (e.g. seed germination and seedling growth, root and root hair growth), reproductive growth (e.g. pollen development and germination, fruit ripening) and responses to biotic (e.g. fungal, viral pathogen, wounding or insect attacks) and abiotic (e.g. cold, heat, salt or drought) stresses (Laohavisit and Davies 2011; Clark *et al.* 2012; Davies 2014; Konopka-Postupolska and Clark 2017). Annexins perform their physiological functions by implicating in Ca^2+^ signalling, metabolism catalysing and vesicle trafficking. Several annexins in maize and *Arabidopsis* have been proved to build reactive oxygen species (ROS)-responsive Ca^2+^ channels, which may be involved in stimuli-induced Ca^2+^ signalling (Laohavisit *et al.* 2009, 2012). Several annexins are involved in cellular redox reaction as peroxidases, suppress stress-induced ROS accumulation, reduce lipid peroxidation and protect cell activity (Zhou *et al.* 2011; Chu *et al.* 2012; Qiao *et al.* 2015; He *et al.* 2019). As membrane lipid- or cytoskeleton-binding proteins, some annexins have been supposed to be involved in cytoplasmic vesicle trafficking and cell secretion (Clark *et al.* 2005; Scheuring *et al.* 2011; Zhu *et al.* 2014; Konopka-Postupolska and Clark 2017). Nevertheless, the role of annexin-mediated vesicle trafficking in certain physiological process needs to be further elucidated. Here, to verify the role of annexins in regulating auxin transport and localization, the role of two annexins, AtANN1 and AtANN2, in unilateral BL-induced phototropism, auxin and auxin transporter distribution in etiolated hypocotyls were investigated. The results showed that, during BL-induced phototropic response, AtANN1 and AtANN2 were involved in inducing asymmetric distribution of PIN3 in etiolated hypocotyls, which led asymmetric distribution of auxin.

## Materials and Methods

### Plant materials

*Arabidopsis thaliana*, including wild type (Col-0) and loss-of-function null mutants of AtANN1 and AtANN2, were used. Two single-null mutants, *atann1* (SALK_132169) and *atann2* (SALK_054223), were both derived by T-DNA insertion and double-null mutant *atann1/atann2* was obtained by hybridization of two single-null mutants and hybrid progeny screening. Seeds were gift from Dr Julia Davies in Department of Plant Sciences, University of Cambridge, UK. *PIN3-GFP* and *DR5-GFP* transgenic Col-0 seeds were gift from Dr Jiří Friml in Institute of Science and Technology Austria, Austria. *PIN3-GFP* and *DR5-GFP* transgenic mutants were obtained by cross-hybridization between *AtANN1* or *AtANN2* null mutant plants and *PIN3-GFP* or *DR5-GFP* transgenic Col-0 plants and hybrid progeny screening.

Seeds were sterilized for 10 min in 75 % (v/v) ethanol, washed twice with deionized water and then sown onto the surface of solid medium consist of 1/2 Murashige and Skoog salt, 1 % sucrose and 0.9 % phytagel at pH 6.0. Square culture dishes were sealed with air-permeable transparent tape. After being stored at 4 °C for 2 days, culture dishes were coated with a foil and transferred into a growth chamber that was maintained at 22 °C.

### Phototropic response detection

Three days after germination, foils were removed and transparent culture dishes were transferred into a chamber which was equipped with blue and red LED (light-emitting diode) light strips, each of which was composed of 30 light beads, on the left-hand side wall. The illumination density was adjusted by the number of initiated light beads and measured using an illumination meter (TES-1339P, TaiShi, Taiwan, China). The wavelength range of BL and RL was 465–475 nm (BL) and 620–630 nm (RL), respectively. Three unilateral illumination combinations (blue, red and blue + red) were used. Culture dishes were vertically placed and in parallel with the illumination direction. The chamber was running under 22 °C, relative humidity 70 %.

After being cultured in unilateral illumination for 2, 4 or 6 h, seedlings were photographed with a scanner. Images were analysed using ImageJ software to measure the hypocotyl curvature of etiolated seedlings. Data were statistically analysed using Sigma Plot 5.0 software.

### Confocal laser scanning microscopy

Seedlings of *PIN3-GFP* and *DR5-GFP* transgenic lines were cultured as described above. Three-day-old etiolated seedlings were grown in 1/2 Murashige and Skoog medium, cultured and unilateral illuminated for a certain period of time (see detail in corresponding figures). Seedlings were then imaged using confocal laser scanning microscope (Meta-710, Zeiss, Germany). GFP was excited using a 488 nm argon laser; emitted light was collected through a 525 ± 5.5 nm filter. Captured images were edited using Adobe Photoshop 7.02. Fluorescent intensity was measured by using ImageJ software. Data were statistically analysed using Sigma Plot 5.0 software.

## Results

### Unilateral BL/RL induced phototropism of etiolated hypocotyls of *Arabidopsis thaliana*

**Figure 1.**
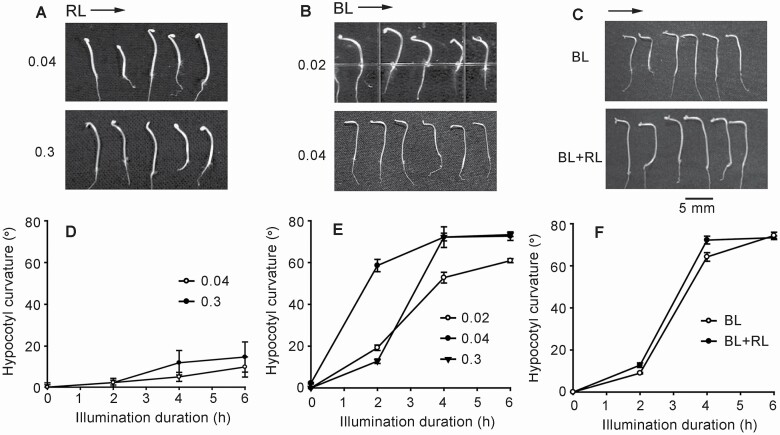
Phototropic response of seedlings to RL and BL. Etiolated *Arabidopsis thaliana* (Col-0) seedlings were illuminated with unilateral RL or BL. (A–C) Images of growing seedlings which were illuminated with unilateral RL (A), BL (B) or RL + BL (C) for 6 h. Illumination intensity (μmol·m^−2^ s^−1^) is marked on the left side of each figure; arrows on the top of each figure mark the direction of illumination. The scale bar is shown on the bottom of (C). (D–F) The hypocotyls’ curvature after illumination. In each experiment, 40–50 seedlings were measured, data from three replicates were calculated to obtain mean ± SE.

To establish how the *A. thaliana* seedlings respond to BL and/or RL under our experimental conditions, etiolated seedlings were illuminated by unilateral RL, BL or RL + BL. The time course for phototropic bend growth was then investigated. Unilateral RL weakly induced phototropic bending. Two hours after illumination with either 0.04 or 0.3 μmol·m^−2^ s^−1^ RL, hypocotyls started to bend, the curvature increased slowly during the following 4 h (Fig. 1A). Hypocotyls’ curvatures were as follows: 3 ± 2.3°, 5 ± 2.4° and 10 ± 5.4° after 2, 4 and 6 h RL (0.04 μmol·m^−2^ s^−1^) illumination; 3 ± 1.3°, 12 ± 6.4° and 15 ± 8.0° after 2, 4 and 6 h RL (0.3 μmol·m^−2^ s^−1^) illumination, respectively (Fig. 1D). Statistical results showed that curvature at 6 h was significantly bigger than that of control (*P* < 0.05).

Unilateral BL strongly induced phototropism. Two hours of illumination induced notably bending, then curvature gradually increased over the following 4 h and reached its peak value at 6 h (Fig. 1B). Hypocotyls’ curvatures were as follows: 19.2 ± 1.3°, 52.8 ± 2.6° and 61.0 ± 0.9° after 2, 4 and 6 h BL (0.02 μmol·m^−2^ s^−1^) illumination; 58.6 ± 1.5°, 72.1 ± 2.5° and 72.6 ± 1.0° after 2, 4 and 6 h BL (0.04 μmol·m^−2^ s^−1^) illumination; 12.8 ± 1.1°, 72.2 ± 1.9° and 73.4 ± 0.9° after 2, 4 and 6 h BL (0.3 μmol·m^−2^ s^−1^) illumination, respectively. All three BL intensities significantly induced phototropic response (*P* < 0.05) (Fig. 1E).

To verify whether RL can promote BL-induced phototropism, etiolated seedlings were illuminated by unilateral BL (0.3 μmol·m^−2^ s^−1^) + RL (0.3 μmol·m^−2^ s^−1^). Comparing with control (BL only), RL + BL-induced phototropic bending was only a little stronger (Fig. 1C). Two, four and six hours after BL illumination, hypocotyls’ curvatures were 9 ± 0.4°, 64.2 ± 2.0° and 74.4 ± 1.6°; BL + RL-induced hypocotyls’ curvatures were 12.8 ± 1.1°, 72.2 ± 0.8° and 73.3 ± 1.9°, respectively (Fig. 1F). Given these results, RL did not significantly promote BL-induced phototropic response (*P* > 0.05).

Above all, in this experimental condition, unilateral BL/RL induced phototropism of etiolated hypocotyls of *A. thaliana*, and BL-induced response was much stronger than RL. Since added RL did not significantly promote BL-induced phototropism, in the following experiments, only BL was used as a unilateral stimulation.

### AtANN1 and AtANN2 are involved in BL-induced phototropism of hypocotyl

**Figure 2.**
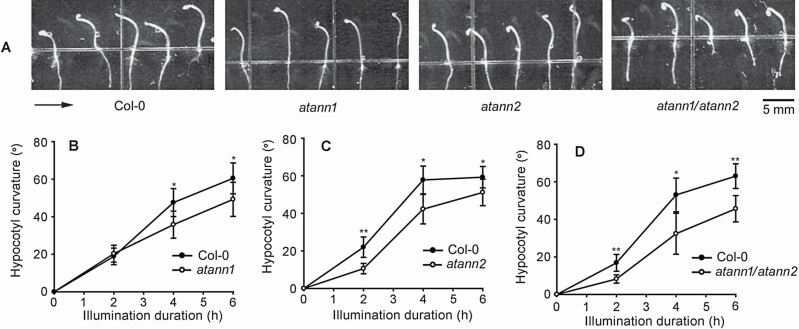
AtANN1 and AtANN2 are involved in hypocotyls’ phototropic response to BL. Etiolated seedlings of single- or double-null mutant were illuminated with unilateral BL (0.02 μmol·m^−2^ s^−1^). (A) Images of seedlings that were illuminated for 6 h. The arrow marks the direction of illumination. The scale bar is shown at the bottom-right corner. (B–D) Hypocotyls’ curvature after illumination. In each experiment, 40–50 seedlings were measured, data from three replicates were calculated to obtain mean ± SE. Student’s *t*-test *P*-values: *P < 0.05, **P < 0.01.

To verify the role of AtANN1 and AtANN2 in BL-induced phototropism, response of *AtANN1* and *AtANN2* single- or double-null mutant seedlings to unilateral BL (0.02 μmol·m^−2^ s^−1^) was investigated. Etiolated hypocotyls of *atann1* and *atann2* responded to BL as marked bending, which was appearing 2 h after illumination. Their hypocotyls’ curvature increased gradually, reaching their peak at 6 h (Fig. 2A). However, comparing with wild type, two mutants showed weakened responsiveness to BL. Two, four and six hours after illumination, hypocotyls’ curvatures of *atann1* were 20.3 ± 4.5°, 35.7 ± 7.2°, 49.2 ± 9.1°, of *atann2* were 10.6 ± 2.8°, 42.2 ± 7.9° and 51.1 ± 7.0°, both of which were significantly smaller than those observed in wild type (*P* < 0.05) (Fig. 2B and C). Two, four and six hours after illumination, hypocotyls’ curvatures of *atann1/atann2* were 8.2 ± 2.2°, 32.3 ± 10.9° and 45.7 ± 7.0°, which were markedly smaller than those observed in wild type or in the two single-null mutants (*P* < 0.05) (Fig. 2D).

### AtANN1 and AtANN2 are involved in unilateral BL-induced asymmetric auxin distribution in hypocotyls

**Figure 3.**
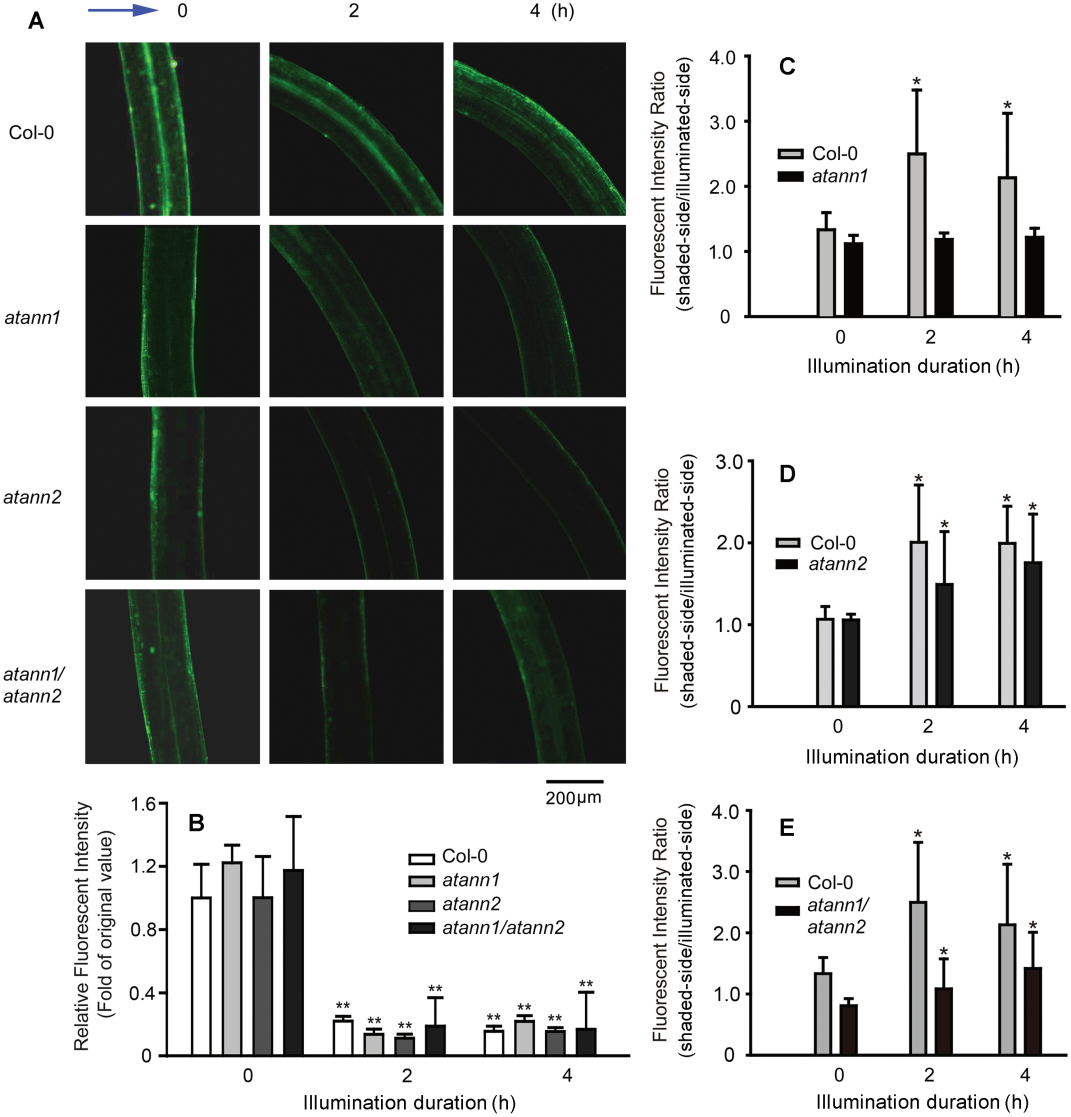
AtANN1 and AtANN2 are involved in unilateral BL-induced asymmetric auxin distribution in hypocotyl cells. *DR5-GFP* transgenic line seedlings were illuminated by unilateral BL (0.02 μmol·m^−2^ s^−1^). (A) DR5-GFP fluorescence in hypocotyl cells. The blue arrow at the left upper corner marks the direction of unilateral BL. The numbers on the top of the image note illumination duration. The scale bar is showed on the bottom-right corner. (B) Fluorescent intensity in hypocotyls. (C–E) Fluorescent intensity ratio (shaded side/illuminated side) after illumination. In each experiment, 40–50 seedlings were measured, data from three replicates were calculated to obtain mean ± SE. Student’s *t*-test *P*-values: *P < 0.05, **P < 0.01.

To verify the mechanism behind annexins-mediated phototropic response, *DR5-GFP* transformant (of Col-0) etiolated seedlings were illuminated by unilateral BL (0.02 μmol·m^−2^ s^−1^), fluorescence was monitored as a proxy for auxin distribution in hypocotyl cells. After 2 h of BL illumination, mean fluorescent intensity in hypocotyl cells decreased (Fig. 3A), mean fluorescent intensity was significantly lower than control (*P* < 0.05) (Fig. 3B). Meanwhile, a marked asymmetric distribution of fluorescence in hypocotyls appeared, with fluorescent intensity in epidermal cells of the illuminated side weaker than the shaded side. Fluorescent intensity ratios (shaded side/illuminated side) were 2.01 ± 0.7 at 2 h and 1.99 ± 0.45 at 4 h, respectively, both of which were significantly higher than control (*P* < 0.05) (Fig. 3C).

To verify the role of AtANN1 and AtANN2 in BL-affected auxin distribution, etiolated seedlings of *DR5-GFP* transformants of *atann1*, *atann2* or *atann1*/*atann2* were illuminated by unilateral BL (0.02 μmol·m^−2^ s^−1^), fluorescence was detected as a proxy for auxin distribution. Two hours after illumination, fluorescent intensity in hypocotyls of the three transformants decreased markedly (Fig. 3A). Comparing with Col-0, fluorescent intensity decrement in the three mutants was not significantly different (Fig. 3B). However, fluorescent intensity ratio (shaded side/illuminated side) in etiolated hypocotyls of *DR5-GFP* transformants of *atann1*, *atann2* or *atann1/atann2* was all significantly lower than that in Col-0 (*P* < 0.05) (Fig. 3C–E).

### AtANN1 and AtANN2 are involved in unilateral BL-induced asymmetric distribution of PIN3 in hypocotyl cells

**Figure 4.**
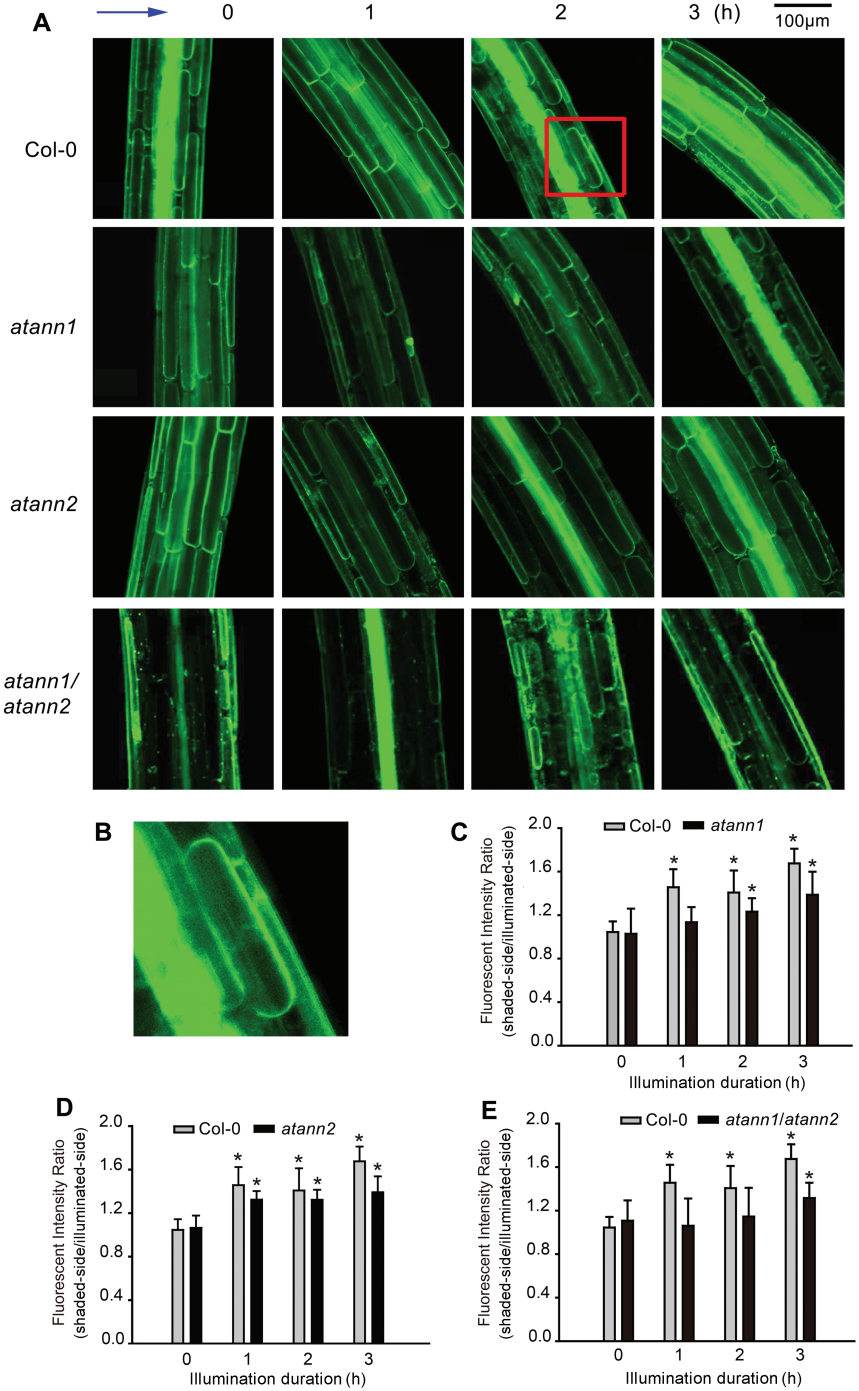
AtANN1 and AtANN2 are involved in unilateral BL-induced asymmetric distribution of PIN3 in hypocotyl cells. *PIN3-GFP* transformant seedlings were illuminated by unilateral BL (0.02 μmol·m^−2^ s^−1^). (A) PIN3-GFP fluorescence in hypocotyl cells. The blue arrow at the left upper corner marks the direction of unilateral BL. The numbers on the top of the image note illumination duration. The scale bar is showed on the upper-right corner. (B) An enlarged view of the image in the red box in (A). (C–E) Fluorescent intensity ratio (shaded side/illuminated side) after illumination. In each experiment, 40–50 seedlings were measured, data from three replicates were calculated to obtain mean ± SE. Student’s *t*-test *P*-values: *P < 0.05.

To verify the mechanism of annexins-mediated auxin asymmetric distribution in BL-induced tropic response, etiolated seedlings of *PIN3-GFP* transformants of Col-0, *atann1*, *atann2* and *atann1/atann2* were illuminated by unilateral BL (0.02 μmol·m^−2^ s^−1^) and fluorescent intensity in etiolated hypocotyls was detected. Two hours after illumination, a marked asymmetric distribution of PIN3-GFP in epidermal or endodermal cells appeared. In individual cells, fluorescent intensity in PM on the illuminated side was remarkably weaker than that on the shaded side (Fig. 4A and B). In Col-0, fluorescent intensity ratio (shaded-side PM/illuminated-side PM) was significantly increased after BL illumination (Fig. 4C–E). In *PIN3-GFP* transformants of *atann1*, *atann2* and *atann1/atann2*, the asymmetric distribution of PIN3-GFP was induced by BL as well, however, notably weaker than that observed in wild type. In *atann1*, *atann2* and *atann1/atann2* hypocotyls, 1, 2 and 3 h after BL illumination, fluorescent intensity ratios were all significantly lower than those in wild type (*P* < 0.05) (Fig. 4C–E).

## Discussion

Tropic growth allows plants to acquire favourable factors and avoid harmful ones in their surroundings. It has been shown that phototropism is stimulated mainly by BL, RL is involved to a less extent as well (Kagawa *et al.* 2009; Liscum *et al.* 2014; Haga *et al.* 2015). Here, the feature of phototropic response of etiolated seedlings to individual blue/red light or blue + red light (Fig. 1), including the illumination intensity dependence and time course of subsequent phototropic bending were consist to reported results, in which 0.0017–0.17 μmol·m^−2^ s^−1^ BL induced bend growth of hypocotyls (Haga *et al.* 2014, 2015). Since RL only slightly induced hypocotyl bending, we focused on BL-induced responses in the following work.

In *A. thaliana* plants, AtANN1, AtANN2, AtANN3 and AtANN4 are abundant in hypocotyls and young stems (Clark *et al.* 2001, 2005). In our preliminary work (data not shown), of the eight annexins in *Arabidopsis*, *AtANN1* and *AtANN2* null mutants showed decreased responsiveness to BL. Given this, the roles of AtANN1 and AtANN2 in hypocotyls’ phototropism were intensively investigated. Comparing with Col-0, seedlings of *atann1* or *atann2* showed weakened responsiveness to BL, with slower responding speed and small hypocotyl curvature. In *atann1/atann2* seedlings, the responsiveness was further decreased. The data preliminarily indicate the possible involvement of AtANN1 and AtANN2 in BL-induced phototropism. Thus far, there remain no reports regarding the role of any annexin in the phototropic responses. Given this, the results provided here offer new clues towards a better understanding of the role of annexins in plant growth regulation. Further results indicated that lost-of-function of *AtANN1* and *AtANN2* led the decrease or impairment of unilateral BL-induced asymmetric distribution of PIN3, so we suggest that the two annexins may participate BL-induced transport or circulation of PIN3, and then auxin lateral transport may lead its asymmetric distribution, which will finally induce bending of etiolated hypocotyl.

Auxin plays essential roles in plant tropism. Asymmetric auxin distribution is the main reason for most of stimuli-induced bending (Fankhauser and Christie 2015; Harmer and Brooks 2018; Han *et al.* 2021). Several PINs and ABCB19 are involved in light/gravity-induced auxin asymmetry (Kleine-Vehn *et al.* 2010, 2011; Liscum *et al.* 2014). In hypocotyls, PIN3 plays central role in stimuli-induced auxin transport. Responding to light or gravity, the abundance and localization of PIN3 changed and then altered auxin transport, leading asymmetric distribution of auxin which will finally change the growth direction of hypocotyls (Friml *et al.* 2002; Ding *et al.* 2011; Rakusová *et al.* 2011). Nevertheless, the mechanism behind BL-induced PIN3 relocation remains unclear. The results here, in which two membrane-associated proteins (AtANN1 and AtANN2) are involved in unilateral BL-induced auxin asymmetric distribution and PIN3 relocalization, provide new clues to clarify the mechanism.

AtANN1 has been reported to be involved in stimuli-triggered Ca^2+^ signalling by mediating Ca^2+^ influx as ROS-activated Ca^2+^ channels (Laohavisit *et al.* 2012, 2013; Wang *et al.* 2015). AtANN1 and AtANN2 are involved in post-phloem sugar transport in root tips as well (Wang *et al.* 2018). Most recently, it was proved that in epidermal cells of several plant organs (including hypocotyls), AtANN1 enriched in PM and vesicles, indicating that it may be involved in vesicle formation and trafficking (Ticha *et al.* 2020). Blue light stimulates Ca^2+^ influx from apoplast or release from intracellular organelles (Harada *et al.* 2003; Stoelzle *et al.* 2003). Nevertheless, the role of BL-stimulated Ca^2+^ signalling in phototropism remains unclear, and the channels in PM which charge BL-stimulated Ca^2+^ influx need to be verified as well. Blue light stimulates accumulation of ROS in plant cells (Consentino *et al.* 2015), the role of such an effect in BL-induced phototropism remains unclear. Unilateral BL-stimulated vesicle trafficking has been proposed to be involved in auxin transporter circulation and subsequent auxin asymmetric distribution (Titapiwatanakun and Murphy 2009; Ding *et al.* 2011; Kleine-Vehn *et al.* 2011; Nishimura *et al.* 2012). AtANN1 and AtANN2 have been reported to be involved in generating Ca^2+^ signalling, reducing ROS accumulation and participating vesicle trafficking (Davies 2014; Konopka-Postupolska and Clark 2017; Ticha *et al.* 2020), all the three physiological processes are possibly mediated BL-induced responses. The mechanism of annexins-mediated phototropic responses needs to be carefully investigated before we can draw a clear conclusion.

## Supporting Information

The following additional information is available in the online version of this article—

File S1. The raw data.

plab075_suppl_Supplementary_Data

## Data Availability

The raw data are available as **Supporting Information**.

## Notes

**Phenome, Genome & Environment. Chief Editor**: Colleen Doherty
